# Salidroside alleviates diet‐induced obesity and insulin resistance by activating Nrf2/ARE pathway and enhancing the thermogenesis of adipose tissues

**DOI:** 10.1002/fsn3.3450

**Published:** 2023-06-06

**Authors:** Xiaozhen Zhu, Ting Ren, Qiushuang Xiong, Zhengfeng Lin, Xiaoxiao Lin, Guangyong Lin

**Affiliations:** ^1^ Department of Pharmacy The Second Affiliated Hospital & Yuying Children's Hospital of Wenzhou Medical University Wenzhou China; ^2^ College of Life and Environmental Science Wenzhou University Wenzhou China

**Keywords:** insulin resistance, Nrf2, obesity, salidroside, thermogenesis

## Abstract

Recent reports suggest that salidroside protects cardiomyocytes from oxidative injury and stimulates glucose uptake by skeletal muscle cells. Despite these findings, the therapeutic potential of salidroside in the treatment of obesity and insulin resistance remains uncertain and requires further investigation. In the present study, the treatment effect of salidroside on the onset and development of the obese phenotype and insulin resistance as well as the underlying mechanisms was investigated using long‐term high‐fat diet‐induced obese mice supplemented with salidroside. We used biochemical kits to determine serum biochemical parameters (including triacylglycerol, total cholesterol, high‐density lipoprotein cholesterol, low‐density lipoprotein cholesterol, fasting glucose, and insulin). The results show that salidroside‐supplemented animals showed better glucose tolerance and insulin sensitivity, decreased blood lipids, and weight gain (*p* < .05). Protein expression of p‐Nrf2 and Nrf2 was analyzed by western blotting, and the mRNA levels of thermogenic‐related genes (*Ucp1*, *Pgc1a*, *Prdm16*, and *Cidea*) were detected by quantitative RT‐PCR. The results show an improvement in lipid peroxidation and Nrf2/ARE signaling, as well as an increased expression of the *Ucp1*, *Pgc1a*, *Prdm16*, and *Cidea* (*p* < .05). Our evidence suggests that salidroside alleviates diet‐induced obesity and insulin resistance potentially by activating Nrf2/ARE pathway and enhancing the thermogenesis of adipose tissues. This induction represents a potential technique for the management of comorbidities related to obesity and its prevention.

## Graphical abstract

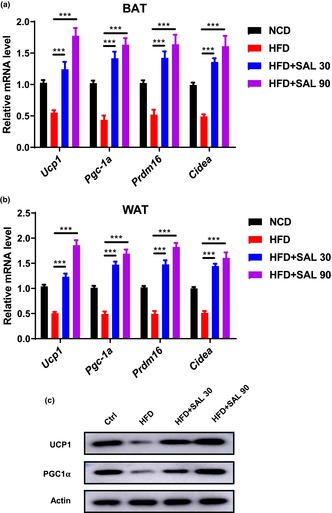

## 1 INTRODUCTION

Obesity is caused by a chronic imbalance between energy intake and expenditure (Liu et al., 2020). Obesity is an important risk factor for a variety of metabolic diseases, such as type 2 diabetes and hyperlipidemia (Sharma et al., 2019). Energy intake exceeds energy expenditure when obesity occurs. Effective treatment strategies for obesity should aim to either decrease energy intake, increase energy expenditure, or a combination of both approaches (Tseng et al., 2010). Accumulated evidence indicates that altering the metabolic efficiency of energy utilization and increasing energy expenditure within adipose tissue are promising strategies to prevent obesity (Sun & Qu, 2019; Xie et al., 2017; Xu et al., 2020).

White adipose tissue (WAT) and brown adipose tissue (BAT) are two distinct types of adipose tissue that play different physiological roles in mammals (Cheng et al., 2021). WAT is characterized by white adipocytes containing large unicameral lipid droplets, white adipocyte store excess energy as triglyceride (TG). BAT is composed of adipocytes with multilocular lipid droplets and numerous mitochondria containing uncoupling protein 1 (UCP1) (Negron et al., 2021). Through a process known as nonshivering thermogenesis, BAT actively contributes to energy expenditure, which dissipates chemical energy to fend off hypothermia and obesity (Zhang, Yang, et al., 2021). BAT activity has been linked to body weight in recent research, and pharmacologically boosting BAT activity and development may be a viable way to combat metabolic disorders (Singh et al., 2021).

Reactive oxygen species (ROS), which are produced during oxidative stress, play a crucial role in the etiology of metabolic disorders, according to studies (Jat & Nahar, 2017). The Nrf2/ARE pathway is crucial to the body's ability to maintain redox homeostasis. According to research, the Nrf2/ARE pathway is activated and regulated by phosphatidylinositol kinase (PI3K), protein kinase C (PKC), mitogen‐activated protein kinases (MAPKs), and other significant intracellular signaling pathways (Saha et al., 2020).

Salidroside, a phenylpropanoid glycoside compound, is the active ingredient of the root of *Rhodiola rosea* (Zhang, Xie, et al., 2021; Zhang, Yang, et al., 2021). According to recent studies, salidroside improves insulin sensitivity in high glucose‐treated hepatocytes (Zheng et al., 2018) and alleviates diabetic neuropathic pain (Zheng et al., 2021). The study reported that salidroside protects cardiac function in mice with diabetic cardiomyopathy (Li et al., 2021). Salidroside consumption could alleviate diabetic nephropathy in rats, the main mechanisms are anti‐inflammatory, antioxidative stress, and inhibition of the TGF‐β1/Smad2/3 pathway (Shan et al., 2021). According to studies, salidroside protects against cardiomyocyte apoptosis and ventricular remodeling in diabetic mice, this cardioprotective effect of salidroside is dependent on AKT signaling activation (Ni et al., 2021). By turning on the AMPK pathway, it can also encourage skeletal muscle cells to take up glucose (Han et al., 2008). However, salidroside's precise effects and mechanisms on insulin resistance and obesity have not yet been documented.

On the basis of the aforementioned facts, we did a systematic exploration in mice, we examined the effects (including body weight, blood lipids, glucose tolerance, and insulin sensitivity) of salidroside on diet‐induced obesity (DIO) mice and its underlying processes. It is hoped that our research will offer a fresh treatment option for diet‐induced obesity and insulin resistance.

## 2 MATERIALS AND METHODS

### 2.1 Animal procedures—Dosage regimen

C57BL/6J mice (male, 8 weeks of age, specific pathogen free) were obtained from the Laboratory Animal Center of Wenzhou Medical University. Mice were given free access to water and food throughout the experiment. Animals were maintained in an animal facility (SPF) at 20–24°C under 50%–60% relative humidity and a 12‐h light/12‐h dark cycle. The experimental design was approved by the Animal Experiment Committee of Wenzhou Medical University (wydw2022‐0916), and the ethical guidelines described in the committee's guidelines for the care and use of laboratory animals were followed throughout the experiments.

Mice were allowed to adapt to this environment for 1 week, and the normal‐chow diet‐fed mice (NCD, *n* = 8) were fed an ad libitum diet (protein: 10.2%; carbohydrate: 67.3%; fat: 6.3%; and other, 2.71 kcal/g) of standard lab chow. Then, another 24 mice fed a high‐fat diet (HFD) (protein: 19%; carbohydrate: 50%; fat: 18%; and other, 4.4 kcal/g) were divided into three groups: the HFD group and two salidroside treatment groups (*n* = 8/group). The treatment groups were treated with salidroside (purity >98%, National Institute for Food and Drug Control, Beijing, China) at a dose of 30 mg/kg (HFD + SAL 30) or 90 mg/kg (HFD + SAL 90) by oral gavage once daily for 10 weeks while the mice were maintained on the HFD.

Body weight and food intake were monitored every week. Glucose tolerance test (GTT) was performed at the end of the animal experiment (week 10). Briefly, mice fasted for 6 h in the morning and then injected intraperitoneally (i.p.) with glucose (2 g/kg·b.w. in saline). Blood samples were collected from the tail vein, and glucose levels were measured using a glucometer (Glucocard SM; Menarini) at 0, 15, 30, 60, and 120 min postinjection. The area under the curve (AUC) for GTT was calculated using GraphPad Prism software. One day after GTT, the mice were deeply anesthetized with CO_2_ suffocation, and blood samples were collected from the orbital sinus and stored in a tube containing EDTA (1 mM). After centrifugation at 1200 *g*, for 10 min, serum was aliquoted and stored at −80°C. Mice were then killed by CO_2_ inhalation before dissection, and brown adipose tissue and gonadal white adipose tissue were harvested and stored at −80°C.

### 2.2 Metabolic and biochemical analysis

Serum biochemical parameters, including triacylglycerol (TG), total cholesterol (T‐CHO), high‐density lipoprotein cholesterol (HDL‐c), low‐density lipoprotein cholesterol (LDL‐c), fasting glucose, and insulin were determined using biochemical kits (A110‐1‐1, A111‐1‐1, A112‐1‐1, A113‐1‐1. Nanjing Jiancheng Bioengineering Institute). The activities of superoxide dismutase (SOD) and catalase (CAT), malondialdehyde (MDA), reduced glutathione (GSH), and oxidized glutathione (GSSG) in gonadal adipose tissue were determined using biochemical kits (A001‐1‐2, A007‐1‐1, A003‐4‐1, A006‐2‐1, A061‐1‐2. Nanjing Jiancheng Bioengineering Institute). The content of 4‐HNE in serum was detected using ELISA kits (Elabscience) and calculated by standard curve. The results were standardized by the total protein concentration measured with the PierceTM Bicinchoninic Acid Protein Assay Kit (Thermo Scientific). The homeostasis model assessment‐insulin resistance (HOMA‐IR) index was calculated as previously described: HOMA‐IR = [(FBG × FINS)/22.5] and the homeostasis model assessment of β‐cell index [(HOMA‐β) = (20 × FINS)/(FBG − 3.5)] (Matthews et al., 1985).

### 2.3 RNA isolation and quantitative RT‐PCR

Total RNA was isolated from frozen tissues using TRIZOL Reagent™ Solution (AM9738; Thermo Fisher Scientific), followed by DNaseI treatment (K2981; Thermo Fisher Scientific). cDNA was synthesized from 1 μg of total RNA using the High‐Capacity cDNA Reverse Transcription Kit (4368814; Thermo Fisher Scientific). Amplification was performed on an ABI PRISM 7700 Sequence Detection System (Thermo Fisher Scientific) under the following reactions: 95°C for 15 min, followed by 40 cycles at 95°C for 10 s, 60°C for 20 s, and 72°C for 20 s. The relative mRNA levels of target genes were normalized to the expression of β‐actin calculated using the 2^−ΔΔ*C*t^ method. The primer pairs used in this study are listed in Table 1.

**TABLE 1:** Designed primer sets for qRT‐PCR.

Gene	Accession number (NCBI)	Primer	5′–3′	Product size (bp)
*Ho‐1*	NM_001132886	Sense	CAGACAGAGTTTCTTCGCCAGAGG	129
		Antisense	TGTGAGGACCCATCGCAGGAG	
*Nqo1*	CT010284	Sense	AGGCTGCTGTGGAGGCTCTG	108
		Antisense	GCTCCCCTGTGATGTCGTTTCTG	
*Ucp1*	KR711477	Sense	AGGCTTCCAGTACCATTAGGT	102
		Antisense	CTGAGTGAGGCAAAGCTGATTT	
*Pgc1α*	MK474634	Sense	TTTACGCAGGTCGAACGAAAC	97
		Antisense	GTGGAAGCAGGGTCAAAATCG	
*Prdm16*	NM_001291029	Sense	CCACCAGCGAGGACTTCAC	113
		Antisense	GGAGGACTCTCGTAGCTCGAA	
*Cidea*	KR710637	Sense	TGCTCTTCTGTATCGCCCAGT	105
		Antisense	GCCGTGTTAAGGAATCTGCTG	
*β‐Actin*	LT575466	Sense	CTGAGAGGGAAATCGTGCGTGAC	93
		Antisense	AGGAAGAGGATGCGGCAGTGG	

### 2.4 Western blotting

The 100‐mg gonadal adipose tissue was lysed in 1‐mL radioimmunoprecipitation assay (RIPA) buffer with phenylmethylsulfonyl fluoride (PMSF, P0100; Solarbio Co., Ltd.) and phosphatase inhibitor (K1015; Apexbio) for 30 min. The volume ratio of RIPA buffer, PMSF, and phosphatase inhibitor is 100:1:1. The lytic product was centrifuged at 4°C with 14,000 *g* for 15 min. Equal amounts of proteins (30 μg/well) were loaded into SDS‐PAGE, separated by electrophoresis at 120 V, and transferred on PVDF membranes (Millipore) at 220 mA (Bio‐Rad). After that, the membranes were sealed at room temperature with 5% nonfatty milk for 4 h and incubated overnight at 4°C in an appropriate amount of diluted primary antibodies (anti‐Nrf2 1:500, anti‐phospho‐Nrf2 1:500, anti‐UCP1 1:1000, anti‐PGC1α 1:1000, and β‐actin 1:500) according to the instructions. Then, membranes were washed twice with TBST buffer for 15 min each time, after incubating at room temperature in the second antibody (1:2000) for 2 h, membranes were washed twice again with TBST buffer, each for 15 min. The signals were finally detected on Molecular Immer ChemiDoc XRS System (Bio‐Rad) with an ECL detection kit (Beyotime). The band intensity ratio of the target protein to β‐actin indicates the relative protein expression level.

### 2.5 Data analysis/statistics

All data were presented as the mean ± SD. The Shapiro–Wilk test was applied to assess data distribution using SPSS (Version 21, IBM Corporation). The statistical differences among animal groups were assessed by using a one‐way analysis of variance (ANOVA) followed by Tukey's test at a statistical significance of *p* < .05.

## 3 RESULTS

### 3.1 Salidroside lowers body weight and blood lipids in DIO mice

**FIGURE 1:**
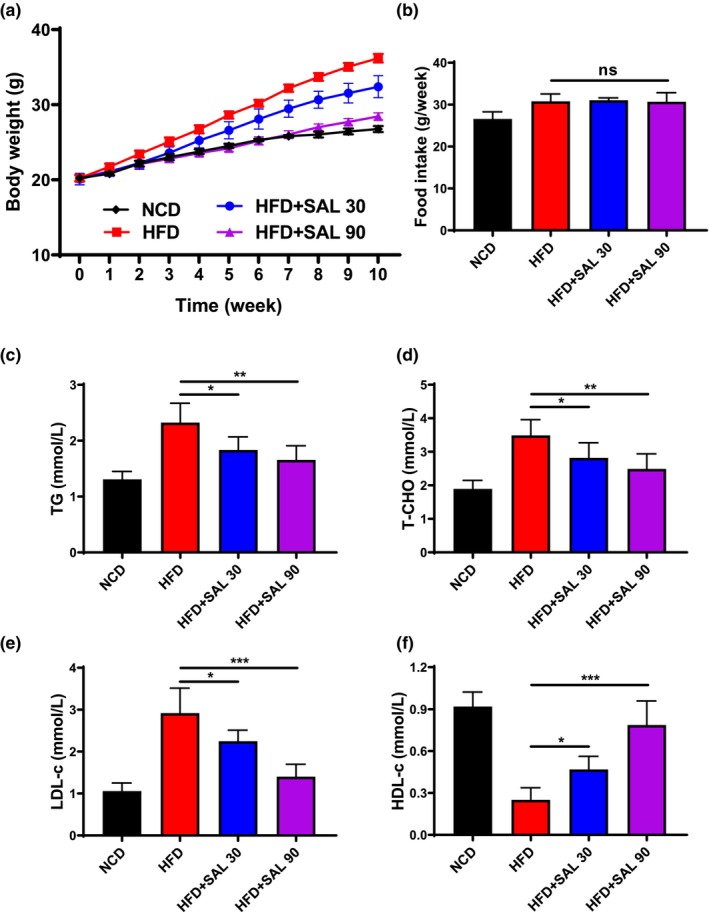
Salidroside reduces high‐fat diet (HFD)‐induced body weight gain and hyperlipidemia. (a) Body weight for the 10‐week intervention with salidroside. (b) Food intake. Plasma levels of (c) triglyceride (TG), (d) total cholesterol (T‐CHO), (e) low‐density lipoprotein cholesterol (LDL‐c), and (f) high‐density lipoprotein cholesterol (HDL‐c). Data are represented as mean ± SD (*n* = 8). *p < .05, **p < .01, and ***p < .001.

To investigate the effect of salidroside on diet‐induced obesity, mice were divided into four groups, fed on a normal chow diet (NCD), high‐fat diet (HFD), HFD supplemented with salidroside (HFD + SAL 30 or HFD + SAL 90) for 10 weeks. The DIO mice fed an HFD (HFD week 10: 36.2 ± 1.39 g) showed apparent weight gain (*p* < .05) compared with the NCD mice (NCD week 10: 26.8 ± 0.99 g), and salidroside treatment (HFD + SAL 30 week 10: 32.4 ± 1.47 g, HFD + SAL 90 week 10: 28.5 ± 1.13 g) significantly reduced the weight gain of HFD‐fed mice (*p* < .05), the effect of salidroside is more pronounced as the dose increases (Figure 1a, *p* < .05). No significant difference was observed in food intake among all experimental groups (Figure 1b, *p* > .05).

To understand whether the HFD induced dyslipidemia, we also tested the serum indicator including triglyceride (TG), serum total cholesterol (T‐CHO), high‐density lipoprotein cholesterol (HDL‐c), and low‐density lipoprotein cholesterol (LDL‐c). The HFD results in a significant increase (*p* < .05) in TG, T‐CHO, and LDL‐c (HFD: TG 2.32 ± 0.35 mmol/L, T‐CHO 3.49 ± 0.47 mmol/L, LDL‐c 2.92 ± 0.60 mmol/L; NCD: TG 1.31 ± 0.14 mmol/L, T‐CHO 1.89 ± 0.25 mmol/L, LDL‐c 1.06 ± 0.19 mmol/L), while salidroside can suppress this effect dose dependently (HFD + SAL 30: TG 1.83 ± 0.23 mmol/L, T‐CHO 2.82 ± 0.45 mmol/L, LDL‐c 2.25 ± 0.26 mmol/L; HFD + SAL 90: TG 1.66 ± 0.25 mmol/L, T‐CHO 2.49 ± 0.45 mmol/L, LDL‐c 1.40 ± 0.30 mmol/L; Figure 1c–e, *p* < .05); the HDL‐c shows lower level in HFD mice (HFD: HDL‐c 0.25 ± 0.09 mmol/L) than NCD mice (NCD: 0.92 ± 0.10 mmol/L), and salidroside dose dependently raised the level of HDL‐c (HFD + SAL 30: 0.47 ± 0.09 mmol/L, HFD + SAL 90: 0.79 ± 0.17 mmol/L; Figure 1f, *p* < .05). The results indicated that mice induced by the HFD were dyslipidemia which was ameliorated by salidroside treatment.

### 3.2 Salidroside improves glucose tolerance and insulin sensitivity in DIO mice

**FIGURE 2:**
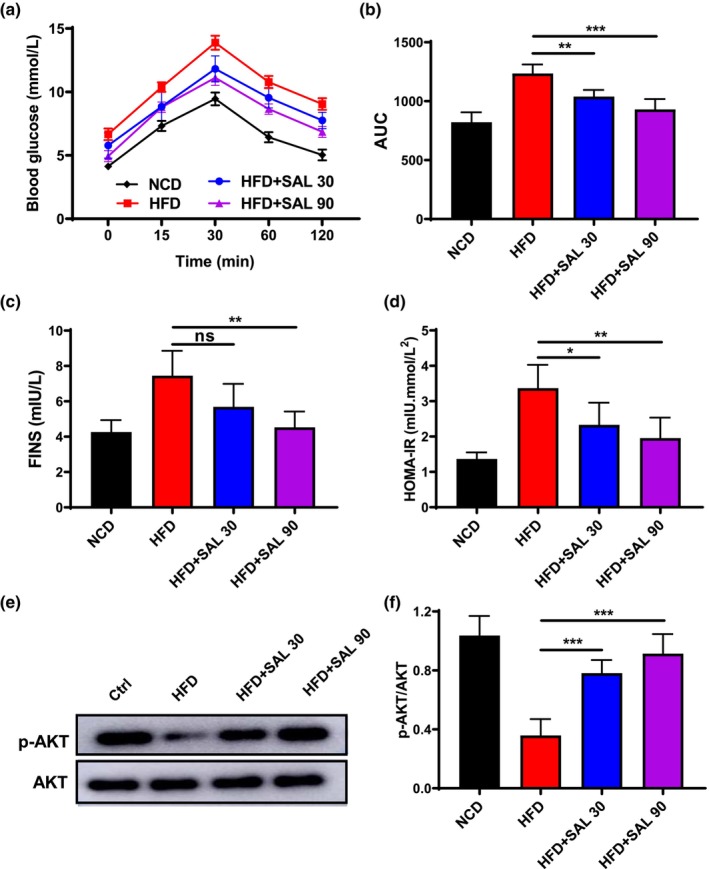
Salidroside ameliorates glucose tolerance and enhances insulin sensitivity in high‐fat diet (HFD)‐fed mice. (a) Glucose tolerance test (GTT) curve showing plasma glucose levels after i.p. administration of glucose (1.5 g/kg b.w.). (b) area under the curve (AUC) of GTT curve (c) fasting insulin (FINS), (d) homeostasis model assessment‐insulin resistance (HOMA‐IR). (e) Protein expression and (f) ratios of p‐Akt and Akt in white adipose tissue (WAT) of HFD‐fed mice analyzed by western blotting and then quantified by densitometry using ImageJ software. Data are represented as mean ± SD (*n* = 8). *p < .05, **p < .01, and ***p < .001.

After the glucose injection, HFD mice show higher concentrations of blood glucose than NCD mice, salidroside can lower blood glucose levels in HFD‐fed mice (AUC: HFD 1235.35 ± 77.02, HFD + SAL 30,1039.84 ± 56.25, HFD + SAL 90,930.33 ± 88.00, *p* < .01, Figure 2a,b). Regarding insulin/glucose responsiveness, we evaluated the fasting insulin (FINS) and homeostatic model assessment of insulin resistance (HOMA‐IR). At week 10 of the salidroside intervention, HFD mice showed a significant reduction in FINS (HFD:7.44 ± 1.42 mIU/L, HFD + SAL 90: 4.53 ± 0.89 mIU/L, *p* < .01, Figure 2c) and HOMA‐IR (HFD: 3.37 ± 0.66 mLU mmol/L^2^, HFD + SAL 30: 2.33 ± 0.62 mLU mmol/L^2^, HFD + SAL 90: 1.96 ± 0.58 mLU mmol/L^2^, *p* < .01, Figure 2d), indicating amelioration of insulin resistance symptoms.

The western blot analysis showed that the protein level of p‐Akt/Akt was significantly decreased by the HFD, and salidroside significantly upregulates the expression level of p‐Akt/Akt protein (HFD:0.36 ± 0.11, HFD + SAL 30: 0.78 ± 0.09, HFD + SAL 90: 0.91 ± 0.13, *p* < .01, Figure 2e,f). Based on these findings, it can be inferred that salidroside upregulation of p‐Akt/Akt expression, thereby improving insulin resistance.

### 3.3 Salidroside alleviates lipid peroxidation through Nrf2/ARE pathway

**FIGURE 3:**
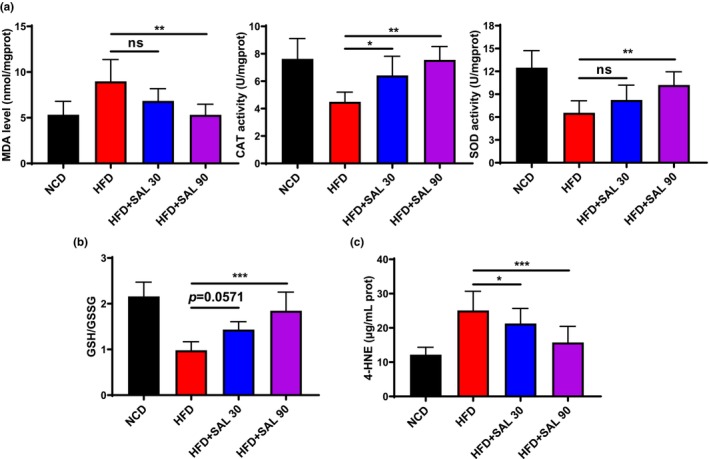
Salidroside ameliorates oxidative stress in white adipose tissue (WAT) of high‐fat diet (HFD)‐fed mice. (a) malondialdehyde (MDA) levels, catalase (CAT), and superoxide dismutase (SOD) activity, (b) reduced glutathione/oxidized glutathione (GSH/GSSG) ratio, and (c) 4‐hydroxynonenal (4‐HNE) levels in WAT. Data are represented as mean ± SD (*n* = 8). *p < .05, **p < .01, and ***p < .001.

Organisms defend against ROS through antioxidant systems, mainly through antioxidant enzymes and nonenzymatic antioxidants. To investigate this protective mechanism, we measured the activity of antioxidant enzymes in mice under different experimental conditions. The HFD mice show higher level of MDA and lower activity level of CAT and SOD. Upon treatment with different doses of salidroside, the activity of CAT and SOD enzymes increased to varying degrees, while the content of MDA decreased. The high‐dose salidroside group had the most significant effect (HFD: MDA 8.98 ± 2.39 nmol/mgprot, CAT 4.50 ± 0.71 U/mgprot, SOD 6.56 ± 1.58 U/mgprot, HFD + SAL 90: MDA 5.31 ± 1.17 nmol/mgprot, CAT 7.55 ± 0.97 U/mgprot, SOD 10.21 ± 1.73 U/mgprot, *p* < .01, Figure 3a). GSH/GSSG is the main dynamic indicator of cellular redox state, the HFD mice show lower GSH/GSSG than NCD mice and salidroside can improve it (HFD: 0.98 ± 0.19, HFD + SAL 90: 1.85 ± 0.41, *p* < .001, Figure 3b). 4‐hydroxynonenal (4‐HNE) is the most representative substance in the aldehyde‐based products of lipid peroxidation, the HFD mice show higher 4‐HNE levels than NCD mice and salidroside can reduce it (HFD: 25.09 ± 5.58 μg/mL prot, HFD + SAL 30: 21.27 ± 4.42 μg/mL prot, HFD + SAL 90: 15.76 ± 4.68 μg/mL prot, *p* < .05, Figure 3c). It means that salidroside can effectively improve the antioxidant capacity of HFD mice.

**FIGURE 4:**
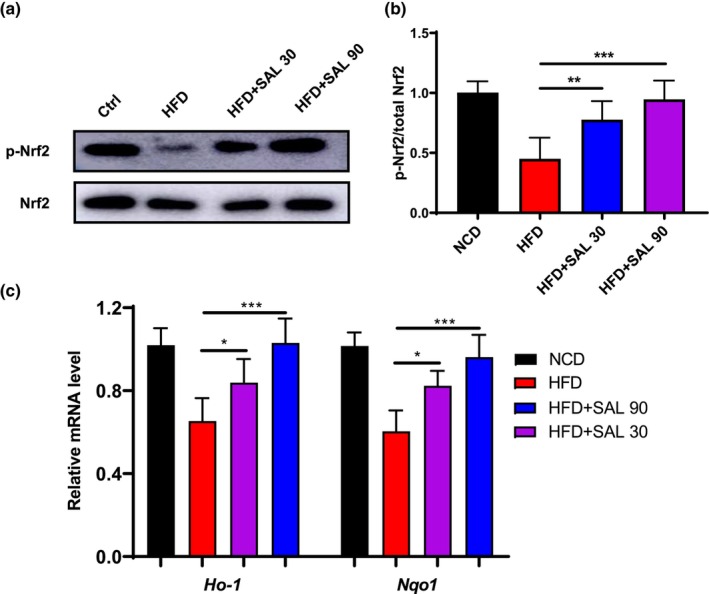
Salidroside activates Nrf2/ARE pathway in white adipose tissue (WAT) of HFD‐fed mice. (a) Protein expression and (b) ratios of p‐Nrf2 and Nrf2 in WAT of high‐fat diet (HFD)‐fed mice analyzed by western blotting and then quantified by densitometry using ImageJ software. (c) Relative mRNA expression of *Ho‐1* and *Nqo‐1*. Data are represented as mean ± SD (*n* = 8). *p < .05, **p < .01, and ***p < .001.

The western blot analysis showed that the protein level of p‐Nrf2/Nrf2 was significantly decreased in the HFD mice, and salidroside significantly upregulates the expression level of p‐Nrf2/Nrf2 protein (HFD: 0.45 ± 0.18, HFD + SAL 30: 0.78 ± 0.16, HFD + SAL 90: 0.95 ± 0.16, *p* < 0.05, Figure 4a,b). Heme oxygenase‐1 (HO‐1) and NAD (P) H: quinone oxidoreductase 1 (NQO1) mainly regulated at the transcriptional level by the nuclear transcription factor Nrf2, the HFD mice show lower *Ho‐1* and *Nqo1* mRNA level than NCD mice, and salidroside can improve it dose dependently (HFD: *Ho‐1* 0.65 ± 0.11 and *Nqo1* 0.60 ± 0.10, HFD + SAL 30: *Ho‐1* 0.84 ± 0.11 and *Nqo1* 0.82 ± 0.07, HFD + SAL 90: *Ho‐1* 1.03 ± 0.12 and *Nqo1* 0.96 ± 0.11, *p* < .05, Figure 4c).

### 3.4 Salidroside enhances nonshivering thermogenesis in BAT and WAT

**FIGURE 5:**
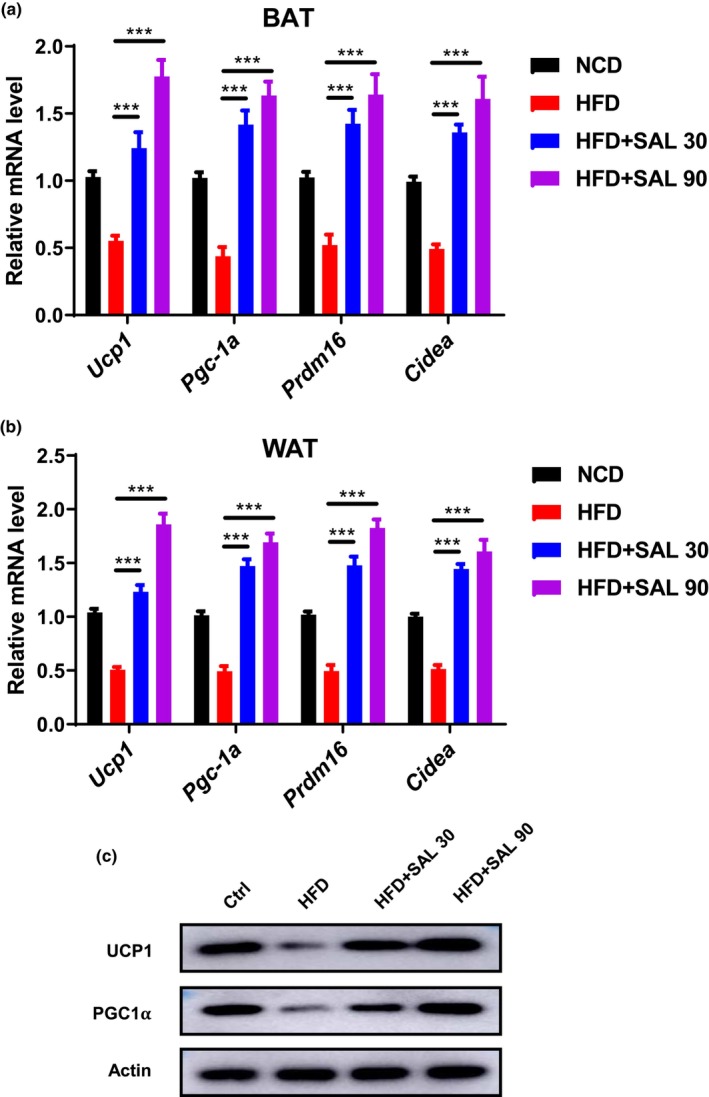
Salidroside induces adipose thermogenesis in HFD‐fed mice. The mRNA levels of thermogenic‐related genes (*Ucp1*, *Pgc1a*, *Prdm16*, *and Cidea*) in (a) brown adipose tissue (BAT) and (b) white adipose tissue (WAT), respectively. (c) The protein expression levels of UCP1 and PGC1α in WAT. Data are represented as mean ± SD (*n* = 8). ***p < .001.

The mRNA levels of genes involved in adaptive thermogenesis, such as uncoupling protein‐1 (*Ucp1*), peroxisome proliferator‐activated receptor gamma coactivator 1 alpha (*Pgc1a*), PR domain containing 16 (*Prdm16*), and cell death inducing DFFA like effector A (*Cidea*), decreased in the BAT and WAT of HFD mice, according to our analysis of salidroside's effects on these The BAT and WAT of HFD mice treated with salidroside also showed higher mRNA levels of *Ucp1*, *Pgc1a*, *Prdm16*, and *Cidea* (HFD: *Ucp1*0.55 ± 0.09, *Pgc1a* 0.44 ± 0.17, *Prdm 16* 0.52 ± 0.19, and *Cidea* 0.49 ± 0.08, HFD + SAL 30: *Ucp1* 1.24 ± 0.29, *Pgc1a* 1.42 ± 0.26, *Prdm 16* 1.42 ± 0.25, and *Cidea* 1.36 ± 0.14, HFD + SAL 90: *Ucp1* 1.78 ± 0.30, *Pgc1a* 1.63 ± 0.25, *Prdm 16* 1.64 ± 0.37, and *Cidea* 1.61 ± 0.40, *p* < .05, Figure 5a,b). Consistent with transcription levels, the salidroside‐treated HFD animals also had higher protein levels of PGC1 and UCP‐1 (Figure 5c). According to these findings, salidroside stimulates energy burning in the BAT and WAT of mice receiving an HFD.

## 4 DISCUSSION

Salidroside treatment significantly decreased the body weight and the levels of TG, T‐CHO, and LDL‐c in the serum of HFD mice, it was suggested that salidroside could enhance HFD‐induced lipid metabolism. Our results are similar to previous reports that salidroside plays a role in reducing adipogenesis (Yang et al., 2020).

Insulin is mainly regulated by the phosphatidylinositol‐3 kinase/protein kinase B (PI3K/Akt) signaling pathway (Hameed et al., 2015). Salidroside may reduce insulin resistance by activating the AMPK/PI3K/Akt/GSK3 pathway, according to recent reports (Zheng et al., 2015). Our results also confirm the similar effects of salidroside regarding insulin resistance. Moreover, salidroside caused a dose‐dependent increase in the phosphorylation of Akt. These findings suggest that the beneficial effects of salidroside on insulin resistance caused by hyperglycemia and hyperlipidemia may be due to the phosphorylation of Akt after salidroside treatment.

Studies have confirmed that obesity and insulin resistance are significantly correlated with the level of oxidative stress, and oxidative stress can inhibit the insulin signaling pathway (Frohnert et al., 2011). Accumulation of ROS has long been thought to lead to insulin resistance (Anderson et al., 2009; Henriksen et al., 2011; Jones & Sies, 2015). High levels of ROS are intricately linked to obesity and associated pathologies, under the conditions of obesity, insulin resistance, T2DM, or hyperlipidemia, ROS production increases, the antioxidant defense system weakens, and the body's oxidative stress level increases (McMurray et al., 2016). Studies have shown that lipid peroxide levels are elevated in the serum of high‐fat‐fed mice (Chen et al., 2022). Our study confirms this and salidroside can dose dependently improve oxidative stress status.

Nrf2 plays a major role in redox homeostasis. When the body is under oxidative stress, redox homeostasis is disrupted, and Nrf2 is released by Nrf2 activators and migrates into the nucleus to bind to antioxidant response elements (AREs), thereby regulating the antioxidant response (Lee et al., 2021). Studies have shown that Nrf2 is ubiquitinated and degraded in the cytoplasm to maintain its normal levels. Oxidative stress inhibits Nrf2 degradation and enhances the nuclear translocation of p‐Nrf2 (Tamami et al., 2019). HO‐1 and NQO1 are potent therapeutic targets for protection against oxidative stress and injury (Yang et al., 2017). According to prior research that supports our current findings, obese model rats' epididymal adipose tissue substantially expressed less *Nrf2* and *Ho‐1* mRNA, which was linked with alterations in TAC, SOD, GSH‐Px, and MDA levels (Zhang et al., 2020). The ability of salidroside to effectively increase Nrf2 and HO‐1 mRNA expression levels and antioxidant activity in epididymal adipose tissue in model rats may explain its protective effect on high‐fat‐induced obesity.

Brown adipose tissue overexpresses UCP‐1 to enhance the uncoupling of mitochondrial respiration, allowing energy to be lost in the form of heat, thereby defending against obesity and related diseases (Porter, 2017). PGC1‐α can promote the browning of WAT by activating a series of transcription factors in PPAR isoforms related to UCP1 transcription (Liu et al., 2018). Studies found that PRDM16 is a key transcription factor that controls the directed differentiation of BAT, which can promote the differentiation of preadipocytes into BAT while inhibiting the formation of WAT (Ji et al., 2019; Wang et al., 2022). *Cidea* is a WAT browning‐related gene. In the current investigation, we discovered that salidroside boosted the expression of several WAT browning‐related genes, which were reported to be downregulated in HFD mice and upregulated in *Ucp1*, *Pgc1a*, *Prdm16*, and *Cidea*. These results imply that salidroside activates PGC1 and UCP1 expression in the WAT, enhancing the browning of the WAT in obese mice.

## 5 CONCLUSIONS

The current work established that the impact of salidroside in lowering body weight and fat mass and enhancing insulin sensitivity in HFD‐induced obese mice was discovered to be connected with activating WAT browning‐related gene expression, specifically those of *Ucp1* and *Pgc1a*. Although additional inquiry is needed on many more concerns involving WAT browning following salidroside therapy, this work presents a promising beginning that leads us to explore the potential role of WAT browning in the action of salidroside.

## AUTHOR CONTRIBUTIONS

**Xiaozhen Zhu:** Investigation (equal); writing – original draft (equal). **Ting Ren:** Investigation (equal); writing – original draft (equal). **Qiushuang Xiong:** Investigation (equal). **Zhengfeng Lin:** Formal analysis (lead); investigation (supporting). **Xiaoxiao Lin:** Formal analysis (supporting); investigation (supporting). **Guangyong Lin:** Conceptualization (lead); supervision (lead); writing – review and editing (lead).

## 6 ACKNOWLEDGEMENTS

This work was financially by Zhejiang medical and health science and technology program (2020KY647).

## CONFLICT OF INTEREST STATEMENT

The authors declare that they have no conflict of interest.

## Data Availability

The data that support the findings of this study are available from the corresponding author upon reasonable request.
